# Review of Current Treatment Intensification Strategies for Prostate Cancer Patients

**DOI:** 10.3390/cancers15235615

**Published:** 2023-11-28

**Authors:** Sobia Wasim, Jieun Park, Seungyoon Nam, Jaehong Kim

**Affiliations:** 1 Department of Biochemistry, College of Medicine, Gachon University, Incheon 21999, Republic of Korea; 2 Department of Neurology, College of Medicine, Dongguk University, Goyang 10326, Republic of Korea; 3 Department of Genome Medicine and Science, College of Medicine, Gachon University, Incheon 21565, Republic of Korea

**Keywords:** radiotherapy/radiation therapy, ADT, LHRH agonist, treatment intensification, prostate cancer

## Abstract

**Simple Summary:**

Prostate cancer (PCa) affects millions of men worldwide, and the mortality rate for locally advanced or metastasized cancer cases remains high. Despite the development of various therapies and approaches, their efficacies still need to be investigated further. This review summarizes the radiotherapy-centered therapeutic combinations and trials that have been tested for the treatment of localized PCa while providing insights into their efficacy and complications.

**Abstract:**

Prostate cancer (PCa) used to be one of the most common nondermatologic cancers in men that can be treated only with surgery. However, a revolutionary breakthrough came in the 1980s with the introduction of long-acting luteinizing hormone-releasing hormone (LHRH) agonists for the curative treatment of PCa. This paradigm shift contributed to the combined use of androgen deprivation therapy (ADT), chemotherapy, and radiotherapy for the treatment. The latest data highlight the use of treatment intensification (TI), i.e., combined use of radiotherapy (RT) and hormonal or drug treatments, for localized or locally advanced PCa. Indeed, the results of combined modality treatments have shown a reduction in disease-specific mortality and improved overall survival. Although TI seems promising, more research studies are warranted to confirm its efficacy. This review summarizes the latest available outcome results of pivotal trials and clinical studies on the efficacy of TI.

## 1. Introduction

Prostate cancer (PCa) is the second most common cancer and one of the main causes of cancer deaths among men worldwide. According to the GLOBOCAN 2020 report, there were 1,414,259 new PCa cases and 375,304 deaths from PCa [1]. The global prevalence of PCa is driven by advancing age, black race, family history, the sedentary lifestyle of people in industrialized nations, and early onset of urological problems. PCa is a highly diverse disease that exhibits a wide variation in clinical progression and biology. The histological origin of cancer cells plays a pivotal role in the determination of clinical outcome, with acinar adenocarcinomas generally having a better prognosis than those with a ductal origin. Furthermore, African men have poorer overall survival (OS) than white men.

Approximately 80% of patients diagnosed with PCa have prostate-limited localized PCa [2]. Localized PCa is a cancer that is inside the prostate gland and has not yet spread to other body parts. During the early stages, clinically localized PCa is often asymptomatic or may be associated with symptoms. When cancer spreads, it may squeeze the urethra and cause urinary problems such as frequent urination, bloody urine, and pain or burning sensation during urination. Many PCas are detected on the basis of aggregated plasma levels of prostate-specific antigen (PSA), a glycoprotein enzyme that is normally expressed by prostate gland tissue [3]. Recent data from the Cancer of the Prostate Strategic Urological Research Endeavor registry stipulate that despite the initiation of PSA screening, approximately 40% of all new patients present with intermediate-risk localized disease [4]. Through PSA testing, unidentified and tiny tumors that may or may not progress to an advanced stage can be identified. However, this strategy has been shown to lack specificity and therefore leads to overdiagnosis and overtreatment of PCa.

For a locally confined disease, potentially curable therapies are available. The current treatment modalities for localized PCa are radical prostatectomy, radiation therapy (RT) with or without androgen deprivation therapy (ADT), and active surveillance (AS). The discovery of ADT in the early 1940s brought the Nobel Prize in Physiology or Medicine to Charles Huggins in 1966. Androgens promote the growth of PCa cells. ADT, a hormone therapy aimed at reducing androgen levels in the body or blocking their action on cancer cells, can be achieved through surgical removal of the testicles or medical therapies that suppress the production or action of androgens (e.g., luteinizing hormone-releasing hormone (LHRH) agonists and antagonists, which reduce testosterone production, and anti-androgens, which block the androgens on cancer cells). Chronic administration of LHRH agonists decreases serum androgen levels similar to those elicited by castration. Schally and colleagues reported that patients with advanced PCa who were administered daily doses of LHRH agonists experienced a 75% reduction in serum testosterone levels, regularization of phosphatase levels in plasma acid, and, most significantly, substantial alleviation of bone pain from metastasized PCa [5]. In recognition of this groundbreaking work, Schally was awarded the Nobel Prize in Physiology or Medicine in 1977. Administration of LHRH agonists has since become the preferred option for ADT in many countries, particularly the United States. ADT plays a pivotal role in the treatment of men (up to 40%) with PCa at some point during the journey of their treatment, with therapeutics target for men with localized intermediate and high-risk disease, and with alleviating target for patients with advanced/metastatic PCa. However, previous studies reported that despite ADT being highly beneficial in the management of advanced PCa, it is not considered a curative treatment due to tumor resistance. Furthermore, the potential resistance of the tumor to castration led to a high morbidity rate among men with PCa.

RT can be used to treat various types of cancer either alone or in combination with other conventional modalities, including surgery [6], chemotherapy, ADT, and immunotherapy, depending on the specific type and stage of cancer [7]. Approximately 60–70% of cancer patients in Europe and the United States currently receive RT, although this percentage may vary by country [8]. For PCa, RT is one of the treatment options, either alone or in combination with additional treatments, such as surgery or hormone therapy. The history of RT for men with PCa dates back to more than a century [9]; in the late 1950s, a revolutionary work by Malcolm Bagshaw et al. revealed the possibility of RT to cure PCa [9,10,11]. Indeed, RT has been a primary nonsurgical treatment for localized PCa for a long time [9,12]; however, despite its efficacy, approximately one-third of patients with localized PCa experience treatment failure within 5 years of the treatment [13,14]. Such a treatment failure is very likely to be associated with not only known anticipating factors indicating the aggressiveness of PCa, such as PSA, T stage, and Gleason score, but also elements related to the intrinsic radioresistance of tumor or the existence of micrometastatic condition at diagnosis or both [15,16]. The innovation of ADT in the early 1940s marked a significant deviation in the treatment of PCa, leading to a decline in the popularity of RT for this disease. 

**Figure 1:**
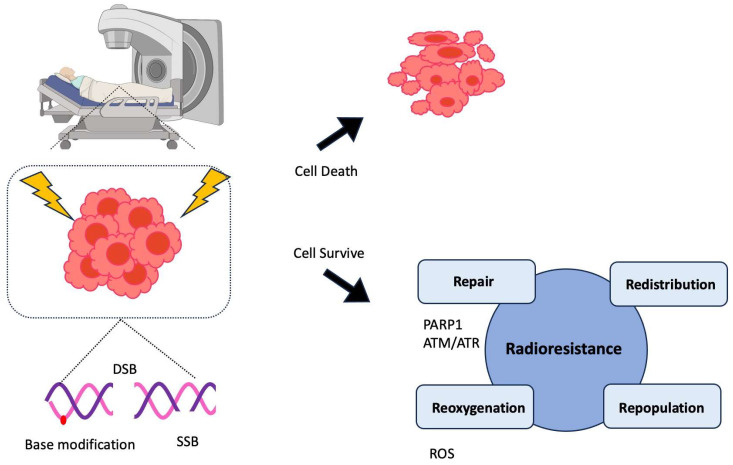
Mechanism of radiotherapy and onset of resistance in prostate cancer (PCa) cells. Radiotherapy (RT), such as external beam RT (EBRT) or high-dose-rate brachytherapy is one of the main treatment options for PCa. The basic principle of this therapy is causing massive and simultaneous DNA damage (double-strand breaks (DSB), single-strand breaks (SSB), and base modifications) that cancer cells cannot recover from, eventually leading to their death. However, in many cases, PCa cells develop resistance to RT through four different mechanisms (4R): repair, redistribution, repopulation, and reoxygenation [19,20]. PARP1 and ATR senses SSB and ATM primarily does DSB. These factors activate downstream signaling pathways and renders cancer cells to survive from DNA toxicity. Aside from repairing DNA lesions, cancer cells can avoid the indirect effect of radiation by redistributing cell cycle, reoxygenizing to hypoxic cells, and rapidly proliferating after IR uptake. These mechanisms help cancer cells survive from IR-induced toxicity.

However, in the 1950s, a renewed interest in RT emerged with the development and availability of high-energy deeper levels perforating cobalt machine [9]. Depending on the location of these radiation sources, RT is mainly divided into external beam RT (EBRT) and brachytherapy. EBRT is the most common type of RT and is especially appropriate for patients with intermediate- or high-risk disease. RT exerts its anticancer effects by damaging the DNA of cancer cells, disrupting the cell cycle, causing cytogenetic damage, promoting apoptosis, and inducing senescence in cancer cells with high-energy ionizing radiation (IR) or photons (Figure 1). These combined effects prevent cancerous cells from proliferating further, ultimately leading to their death [17]. While both normal and cancer cells can be damaged by radiation during cancer treatment, the former are usually able to repair themselves better than the latter [18]. 

Randomized clinical trials confirmed the efficacy of RT for localized PCa, and RT is the mainstay of curative treatment as it effectively targets treatment that destroys cancer cells within the prostate gland [21,22,23]. In recent decades, significant advancements have occurred in the field of RT with the evolution of RT techniques, such as three-dimensional conformal RT and intensity-modulated RT. These advanced radiation techniques can consequently improvise the conformality of high RT dose to the intended treatment area while reducing radiation exposure to normal tissues. This can potentially lead to reduced obstacles and possibly allow a safer dose escalation, which could result in better local disease control. Using these modern technologies, potential phase III dose-escalation trials have displayed a dose response for improvised local and biochemical tumor control [24,25,26,27,28]. In efforts to refine or ameliorate methods for localized PCa, the use of RT with ADT has been scrutinized for many decades [29].

The present study, provides an overview of the latest data supporting the use of RT for localized PCa and highlights the results and findings of randomized clinical trials of RT combined with ADT or RT with ADT and other drugs in various evidence-based treatment combinations for localized PCa. The data were prepared by thoroughly searching the literature from various databases like clinicaltrials.gov, PubMed, web of science.

## 2. Treatment Intensification

PCa is often categorized into risk groups based on several factors, including stage, PSA level, and Gleason score. According to the European Association of Urology guidelines, the commonly used risk categories are (1) low-risk, (2) intermediate-risk, and (3) high-risk groups, which can then be used to help determine treatment of PCa patients.

(1) Low-risk PCa (localized PCa stage T1c or T2a, Gleason score of up to 6, and PSA < 10 ng/mL) is considered less aggressive and slow-growing. Many low-risk patients are potential candidates for AS, which involves regular monitoring rather than immediate treatment. (2) Intermediate-risk PCa (localized PCa stage T2b/c, Gleason score of up to 7, and PSA between 10 and 20) has a moderate risk of progression, and the treatment options include surgery, RT, and AS. (3) High-risk PCa (localized or locally advanced PCa stage T3 or T4; Gleason score of up to 7, 8, or 10; and PSA > 20) is more likely to progress and spread outside of the prostate. 

Treatment options typically include surgery, RT, hormonal therapy, and a combination of these with other drugs. For localized or locally advanced PCa, RT is a curative treatment choice and sometimes used in combination with other treatments, such as ADT and additional agents, to improve treatment outcomes. Because the goal of the treatment is to cure cancer while minimizing aftereffects and preserving the patient’s quality of life, possible options to enhance RT outcomes include increasing the dose of irradiation or reducing the damage to healthy tissues to optimize the radiation effect.

The latest data highlight the application of treatment intensification (TI) for high-risk patients with localized PCa [30]. TI refers to the use of more advanced therapies to manage the disease when standard treatments are insufficient or when PCa recurs. In some cases, TI involves additional RT or ADT to target the recurrent cancer. For patients with high-risk or locally advanced or metastatic PCa, TI may involve both RT and ADT or RT plus ADT with chemotherapies or a combination of these treatments. Indeed, the results of combined modality treatments have shown a decrease in disease-specific mortality and improved OS.

In clinical trials conducted over several decades, two of the most common TI strategies that have been investigated for improving outcomes in patients with localized PCa undergoing RT are RT dose escalation and ADT. Available evidence supports ADT as having a more significant influence on metastasis and PCa-specific mortality (PCSM) than RT dose escalation on unfavorable-risk PCa [31,32].

RT to the primary PCa has emerged as a life-extended element of TI for men with low-volume de novo disease [33]. Despite these successes, how to select patients that are most likely to benefit from the intensification of systemic, local, or metastasis-directed therapy and how to further enhance the efficacy of frontline therapy for metastatic hormone-sensitive PCa (mHSPC) remain unclear. 

When the combination of RT and ADT was compared with RT alone in patients with intermediate- and high-risk PCa, dose-escalation trials have predominantly demonstrated better outcome PSA recurrence rates without survival improvements, such as improved OS, PCSM, and distant metastasis rates. Instead of focusing on the specific radiation dose used, studies that aim to identify patients with a sufficiently high risk of metastasis and PCSM to derive a clinically significant benefit from ADT, as exemplified upcoming NRG GU010 GUIDANCE trial (NCT05050084), are likely represent a more promising approach to optimize the benefits and the risks of ADT in PCa patients undergoing definitive RT. This section discusses various clinical trials that have been attempted over the years to evaluate different treatment therapies for localized or locally advanced PCa as well as their efficacy (Table 1).

**Figure 2:**
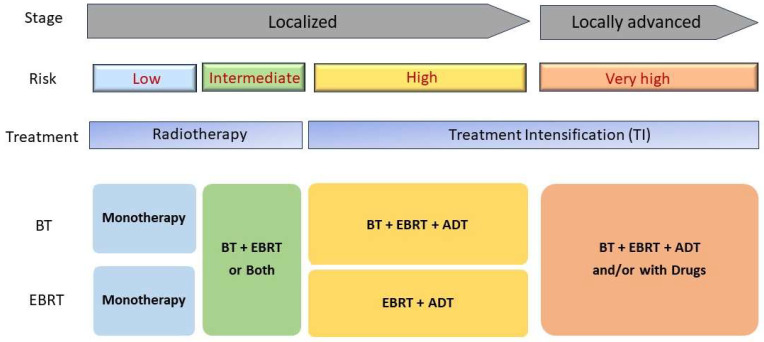
A schematic diagram of treatment intensification for patients with prostate cancers. Localized prostate cancer stage can be categorized into two groups: localized and locally advanced groups. Typical radiotherapy treatment for low-risk localized PC is monoradiotherapy such and brachytherapy (BT) or external-beam radiation therapy (EBRT). For high-risk PC patients, combined treatment of radiotherapy with hormonal therapy so called Androgen deprivation therapy (ADT) clearly shows improved recovery rate.

The treatment strategies may be broadly classified as follows (Figure 2):

### 2.1. RT Alone

Generally, RT is an effective and secure treatment option for localized PCa. RT01 (ClinicalTrials.gov number, ISRCTN47772397), a randomized phase III trial, compared elevated-dose conformal RT with control-dose conformal RT in patients with localized PCa; after 5 years of follow-up, the preliminary findings indicated that elevated-dose conformal RT improved biochemical progression-free survival [28]. NRG Oncology/RTOG 012,6 (NCT00033631), a *phase III trial,* is one of the largest randomized trials that assessed the outcome of radiation dose escalation in localized PCa [34]. 

Dose escalation significantly improved the rates of distant metastases and biochemical control. However, no significant improvement was observed in OS [35]. Thus, dose escalation cannot avoid the need for ADT without randomized evidence [31]. Patients who received high-dose RT experienced more late toxic effects but did not require secondary therapies [34]. Prostate Advances in Comparative Evidence (PACE) is a multicentre, international phase 3 randomized controlled study (NCT01584258) to assess whether hypofractionated stereotactic body radiotherapy (SBRT) [36,37]. SBRT is a rapidly developing treatment option for localized prostate cancer, with good results in terms of local control and toxicity [38,39]. The recent result of PACE-B indicates that high-dose RT modestly improved cancer free rates within a fraction of the standard treatment time and less early toxicity than traditional RT [40].

### 2.2. RT + ADT

The two most common treatment strategies for patients with localized PCa are RT and ADT. However, the ideal duration of ADT remains unclear. Many variations of RT in combination with ADT have been studied, namely, RT with STADT (short-term ADT) and RT with LTADT (long-term ADT), and some trials have even conducted a comparative analysis of these two combinations. With conventional dosing of RT, the addition of ADT improves OS and biochemical recurrence. The incorporation of androgen ablation during, before, and after EBRT has become a standard criterion for treatment. Because dose escalation has also been shown to be beneficial [34], there has been an interest in the use of ADT in the context of dose elevation.

Prolonged ADT can potentially cause significant toxicities, which could impact the patient’s quality of life. Thus, efforts are being made to reduce the duration of ADT to preserve its efficacy. The French-Canadian and Randomized Androgen Deprivation and Radiotherapy (RADAR) PCa Study IV (NCT00223171), a randomized phase III trial, compared ADT for 36 months with ADT for 18 months combined with RT for the treatment of patients with high-risk localized PCa. The findings suggest that the latter is an effective option for localized high-risk PCa, resulting in improved quality of life without compromising survival [41].

The role of RT combined with ADT in the treatment of intermediate-risk PCa remains controversial. Nevertheless, several randomized trials have yielded good results with the different combinations [42,43]; however, these trials were criticized for including patients with different risk stratifications (from low to high risk) and delivery of RT doses that may be considered suboptimal. To examine the hypothesis that dose-escalated RT could either offset or enhance benefit of ADT, a randomized trial involving patients with intermediate-risk PCa was conducted to compare the potential advantage of ADT with two different doses of RT (70 and 76 Gy) versus dose-escalated RT of 76 Gy alone.

Nabid A et al. [44] tested whether dose-escalated RT either negates the benefits of ADT or enhances its efficacy. They conducted a randomized phase III trial (NCT00223145) in patients with intermediate-risk PCa with the aim of comparing the potential benefits between the combination of STADT and RT at a dose of 70 Gy and the combination of ADT and dose-escalated RT of 76 Gy and dose-escalated RT of 76 Gy alone. A median follow-up duration of 11.3 years indicated that in patients with intermediate-risk PCa, the combination of 6-month ADT and either RT at 70 Gy or dose-escalated RT of 76 Gy significantly reduced biochemical failure and alleviated the risk of death from PCa related to dose-escalated RT of 76 Gy alone. Furthermore, a reduction in the radiation dose from 76 to 70 Gy in combination with ADT has been proven to be a viable approach that maintains effective disease control and improves gastrointestinal toxicity profile. A recent study concluded that dose escalation of RT, alone or in the presence of ADT, does not improve metastasis-free survival (MFS), while addition of ADT to RT alone, regardless of RT dose, consistently improves MFS [45]. RT dose escalation showed a high probability of improving biochemical recurrence–free survival.

#### 2.2.1. RT + STADT vs. RT Alone

The combination of RT and STADT has also been demonstrated to improve treatment outcomes in localized PCa. RTOG 94-08 (ClinicalTrials.gov number, NCT00002597), a randomized phase III trial [43], allocated 1979 eligible patients with stage T1b, T1c, T2a, or T2b disease who had PSA level of 20 ng/mL or less to either the RT alone or the RT + STADT group. Patients with intermediate-risk localized PCa who received RT + 4-month STADT compared with those who received RT alone showed a survival benefit. In a median follow-up of 10 years, patients who received RT + STADT had an OS rate of 62% whereas patients who received RT alone had an OS rate of 57%. The addition of STADT decreased the disease-specific mortality from 8% to 4% compared with RT alone. The overall results of the trials indicated that the addition of STADT gave a survival benefit for patients with intermediate-risk PCa who received conventional doses of RT.

GETUG14 (EU-20503/NCT00104741), a multicenter randomized trial involving 377 men with intermediate-risk localized PCa, evaluated the effect of the addition of ADT for 4 months to high-dose RT [46]. The patients were randomly assigned to receive high-dose conformal RT (80 vs. 46 Gy), either conformal RT alone or high-dose conformal RT plus ADT consisting of Triptorelin and Flutamide for 4 months. After a median follow-up of 84 months, it was observed that the group that received both RT and ADT had a significantly higher rate of clinical or biochemical relapse-free survival than the group that received RT alone (84% vs. 76%). Therefore, 4-month androgen blockage combined with RT at an elevated dose was proven to improve event-free survival at 5 years in patients with intermediate-risk PCa [47]. However, no significant difference was observed in OS between the groups (93% vs. 94%). Thus, continued follow-up was necessary to demonstrate any differences in OS. Furthermore, the ADT group more frequently experienced moderate liver toxicity [48]. These studies recommend the use of hormone therapy in combination with RT in intermediate-risk patients.

#### 2.2.2. RT + LTADT vs. RT Alone

EORTC 22863, a randomized phase III trial (NCT00849082) [49], evaluated the benefit of adding LTADT with a LHRH agonist to external irradiation in patients with PCa with an increased risk of metastasis. Patients with T_1–2_ or T_3–4_ PCa were randomly allocated to the RT plus immediate androgen suppression group or the RT-alone group. The results of the 10-year follow-up indicated that treatment with a LHRH agonist with external irradiation improved the disease-free (DFS) and OS of patients with PCa with a high risk of metastasis without increasing the risk for late cardiac toxicity. Bolla et al. [50] previously reported that a randomized phase III trial compared external irradiation alone with external irradiation combined with long-term androgen suppression with a LHRH agonist in locally advanced PCa. The results of a median follow-up of 5.5 years indicated that compared with RT alone, RT plus long-term androgen suppression with LHRH improved the DFS and OS of men with locally advanced PCa.

#### 2.2.3. RT + STADT vs. RT + LTADT 

The combination of EBRT and long-term suppression of androgen (≥2 years) improved OS in men with locally advanced PCa compared with EBRT alone followed by deferral of hormonal treatment until cancer relapse [51,52]. However, prolonged androgen suppression can lower the patient’s quality of life and increase the risk of cardiac infarction [53]. A previous trial (EORTC protocol 22863) that investigated the efficacy of treatment strategies for locally advanced PCa demonstrated that at median accumulation of 3 years, RT + androgen suppression as collate with RT only provided a better benefit with respect to OS [50]. In addition, a recent phase III trial (NCT00003026, EORTC protocol 22863) compared RT + STADT with RT + LTADT for the management of locally advanced PCa. Bolla M. et al. reported that the RT + STADT group had a lower survival rate than the RT + LTADT group [54]. However, both groups experienced adverse effects, including fatigue, decreased sexual function, and hot flashes. 

A phase III DART 01/05 randomized trial (NCT02175212) was performed with patients with clinical stage T1c–T3b N0M0 PCa with high- and intermediate-risk factors to compare STADT (4 months) with LTADT (28 months) in patients treated with high-dose RT [55]. The 5-year results of the DART 01/05 trial indicated that compared with STADT, LTADT + high-dose RT significantly improved MFS, biochemical DFS, and OS in patients with PCa, particularly those with high-risk disease, with no increment in late toxicity [55]. 

Furthermore, Zapatero et al. continued a 10-year follow-up, and the results of the DART 01/05 (NCT02175212) and Eudra CT 2005-000417-36 trials [56] suggested that LTADT does not provide a significant benefit over STADT in patients with intermediate-risk PCa receiving high-dose RT. They did not observe remarkable differences in survival outcomes with LTADT or STADT in the overall cohort or when the patients were analyzed by risk group. However, a more steady and absolute advantage of biochemical DFS, MFS, and cause-specific survival compared with STADT was observed in high-risk patients, suggesting that biological characterization of genomic testing may guide the decision-making process [56]. 

A randomized phase III trial (RTOG 9202 [NCT00767286]) investigated the optimal sequencing of ADT combined with definitive RT in the therapeutic treatment of locally advanced nonmetastatic PCa, This trial assessed 1554 patients with PCa (cT2c–T4, N0–Nx) who had PSA level < 150 ng/mL and who received 4-month ADT during and before EBRT with successive randomization to no additional treatment versus another 2 years of ADT. The trial reported that LTADT was associated with a higher OS rate of 45% compared with STADT, which was 32%, at 10 years [52]. Other previous studies also demonstrated that the addition of RT to LTADT was beneficial in patients with a high risk of cancer recurrence and death from PCa [57,58]. 

The RADAR trial had two hypotheses: (1) that 18-month ADT in combination with RT is a more effective treatment option for locally advanced PCa than 6-month ADT + RT and (2) that 18-month bisphosphonate therapy will avert bone loss caused by ADT and further reduce the adverse effects of androgen suppression and avert bone progression [59]. Zoledronic acid helps avert bone loss during androgen suppression; however, its involvement in the prevention of bone metastases in locally advanced HSPC was unclear. TROG 03.04 (NCT00193856), a randomized trial, explored the effect on PSA control and survival of different continuation of adjuvant ADT in corporation with definitive RT for localized PCa [59]. Although the addition of zoledronic acid to this curation strategy was not useful in preventing bone metastases or other oncological endpoints [60], the 10-year follow-up results of the TROG 03.04 RADAR trial indicated that 18-month ADT + RT was more effective in controlling PSA and improving survival in patients with locally advanced PCa, including high- and intermediate-risk elements, than 6-month ADT + RT. 

However, the optimal ADT duration is still debated when associated with salvage RT. Interpretation of the RTOG-9601 and GETUG-16 results remains obscure; none of these trials being exclusively on the early intervention of post-operative RT with PSA < 0.5 ng/mL [61]. Other factors considered, including pre-existing comorbidities, particularly cardiometabolic disease, and the applicability of radiation dose escalation, may improve estimation of the optimum time period of ADT in combination with RT for locally advanced PCa.

#### 2.2.4. RT + NADT vs. RT Alone

The short-term duration of neoadjuvant ADT (NADT) has been investigated in TROG 96.01 (ACTRN12607000237482), a randomized trial involving 818 patients with locally advanced PCa. [62]. In an open-label study, PCa patients were randomly assigned to the RT alone, RT + 3-month NADT, and RT + 6-month NADT groups. The results of a median follow-up of 10.6 years indicated no significant difference in distant progression, PCSM, and all-cause mortality between the RT-alone and the RT + 3-month NADT groups. However, the RT + 6-month NADT group showed better PCSM and overall mortality than the RT-alone group. This suggests that 6-month NADT combined with RT is an effective treatment for patients with locally advanced PCa, particularly in men without pre-existing metabolic or nodal metastatic comorbidities that could be exacerbated by extended ADT [62]. Another study also showed that NADT initiation 8 to 11 weeks before RT is associated with significantly improved OS compared with shorter NADT duration in high or very high-risk PCa patients [63]. Contrarily, a recent meta-analysis reported that NADT extension from 3–4 months to 6–9 months in patients with localized PCa receiving RT does not improve MFS, indicating that the magnitude of the benefit could vary [64].

#### 2.2.5. Conformal RT + NADT 

RT dose is limited by treatment-related side effects. Conformal RT is a specialized technique developed to more precisely target cancerous tumors while minimizing the side effects [65]. RT01 (ClinicalTrials.gov number, ISRCTN47772397), a randomized phase III trial [28], assigned 862 patients with histologically established T1b–T3a, N0, M0 PCa who had PSA < 50 ng/mL, reported that the higher escalated dose of 74 Gy to the standard dose 64 Gy administered in patients with locally advanced PCa would be safe and effective by use of conformal RT technique. 

The results of a median follow-up of 10 years indicated that elevated-dose conformal RT with NADT improved biochemical progression-free survival but not OS. While this efficacy of escalated-dose treatment is measured against the heightened risk of acute and late toxicities linked to the escalated dose, it emphasizes the significance of using modern RT techniques to reduce the side effects [28].

### 2.3. RT + Immunotherapy

NCT04569461, an ongoing phase 2 trial, is analyzing the trimodality approach of low-dose ionizing radiation with or without neoadjuvant Pembrolizumab, ADT, and prostate SBRT followed by radical prostatectomy for localized prostate cancer (TALON). The primary outcomes measure the percentage of subjects who achieve biochemical progression-free survival (BPFS) at the time frame of 24 months. Another phase 2 randomized clinical trial, NCT03007732, where Pembrolizumab or Pembrolizumab in combination with intratumoral SD-101 (TLR9 agonist) which promotes T cells activation and homing, is being studied in patients with hormone-naïve oligometastatic prostate cancer receiving SBRT and intermittent ADT. It is not yet known whether this trial showed better results in treating patients with PCa.

NCT03543189, another phase 1/2 trials investigated the safety and tolerability of nivolumab when it is given in combination of nivolumab immunotherapy with RT and ADT in the management of Gleason group 5 localized prostate cancer. Youan et al. reported that the combination of nivolumab with ADT and high-dose-rate brachytherapy in patients are well tolerated and associated with evidence of increased immune infiltration and antitumor activity [66]. 

A phase 2 POSTCARD randomized trial (NCT 03795207) was studying the comparative efficacy of Stereotactic Body Radiation Therapy (SBRT) with or without Durvalumab (MEDI4736) in oligometastatic recurrent hormone-sensitive prostate cancer patients. Patients were randomly assigned to either SBRT + Durvalumab (anti PD-L1), or SBRT alone in oligometastatic hormone-sensitive prostate cancer patients. The primary endpoint is two-year progression-free survival and secondary endpoints encompass ADT-free survival, acute and late toxicity, prostate cancer-specific survival, quality of life, overall survival, and immune response [67]. 

NCT03649841, a phase 2 trial, was evaluating the radiation enhancement of local and systemic anti-prostate cancer immune response. However, the clinical trial was terminated early due to poor accrual, leading to small numbers of subject analyzed, and lack of ability to draw scientific conclusion from the low patient numbers.

### 2.4. RT + ADT + Additional Drugs

After clinical trials such as PEACE 1, STAMPEDE, and CHAARTED reported that the addition of other drugs to ADT improved the survival rate in men with mHSPC, many similar clinical trials on localized PCa were initiated. NCT03311555 (STARTAR) (2018–2022), a phase II salvage trial, investigated ADT and an androgen receptor (AR) signaling inhibitor, apalutamide with RT supplemented by docetaxel in patients with PSA recurrent PCa after radical prostatectomy and found the progression-free survival rate increased to 72%. This finding suggests that TI in nonmetastatic PCa is effective and feasible. Analysis of the quality of life of men receiving cancer treatments may help recognize the intermediate- and long-term effects of such treatments on them. Apalutamide is also tested in the randomized phase III CARLHA 2 GETUG 33 trial (NCT04181203), and also in the two-arm phase II NRG GU006 study (NCT03371719) [61]. In a similar approach, DASL-HiCaP: Darolutamide augments standard therapy for localized very high-risk cancer of the prostate (ANZUP1801) is exploring the addition of 96 weeks of Darolutamide to RT and ADT, either for primary definitive therapy or in an adjuvant setting for very high-risk PCa (NCT04136353), and RTOG 3506 (STEEL) is studying the addition of 2 years of Enzalutamide with salvage RT and 2 years of ADT when aggressive features are displayed (NCT03809000) [61,68].

NCT01546987, an ongoing (2012–2029) randomized phase III trial, is analyzing the use of hormone therapy, including TAK-700, in combination with RT. This trial is exploring the variance in the OS of men with clinically localized PCa with adverse prognostic attribute between a) standard treatment [ADT] + RT and b) standard treatment + 24-month steroid 17-alpha-monooxygenase TAK-700. NCT01952223 (PEACE 2), another ongoing trial (2013–2041), is evaluating the benefit of neoadjuvant ADT + Cabazitaxel and pelvic RT in the curative treatment of localized PCa.

**Table 1:** Summary of clinical trials for treatment intensification in patients with localized or locally advanced prostate cancer. Some of the clinical trials on treatment intensification attempted over the years are shown. ‘-’ denotes the outcomes of the study are not clear.

Trials	Status	Study Period	Treatment	Outcomes
NCT00684905: Leuprolide, Bicalutamide, and implant radiation therapy for patients with locally recurrent prostate cancer after external beam radiation therapy	Completed	2000–2005	Leuprolide + Bicalutamide + brachytherapy + Leuprolide	-
NCT00002597: Radiation therapy with or without antiandrogen therapy for patients with stage I or II prostate cancer	Completed	1994–2018	Zoladex + Flutamide + RT	PSA level < 20 ng/mL, use of short-term ADT for 4 months before and during RT was reduced disease-specific mortality and increased overall survival [43]
NCT00016913: Chemotherapy, hormone therapy, and radiation therapy for patients with locally advanced prostate cancer	Completed	2001–2008	Paclitaxel + Estramustine + Carboplatin + Gonadotropin-releasing hormonal therapy (Goserelin/Leuprolide) + RT	The administration of neoadjuvant chemohormonal therapy with TEC, followed by high-dose radiation therapy has demonstrated safety and feasibility [69]
NCT02135445: Safety and efficacy of TAK-385 for patients with localized prostate cancer	Completed	2014–2015	TAK-385 + RT vs. Degarelix + RT	-
NCT00193856: RADAR (randomized androgen deprivation and radiotherapy) trial	Completed	2003–2017	6-month Leuprorelin acetate + RT 6-month Leuprorelin acetate + zoledronic acid + RT 18-month Leuprorelin acetate + RT 18-month Leuprorelin acetate + zoledronic acid + RT	Prostate cancer-specific mortality, Biochemical Failure
NCT00223665: Effects of IAS in men with localized biochemical relapse prostate cancer (IAS)	Completed	1997–2012	RT + intermittent androgen suppression + Flutamide + Leuprolide acetate	-
NCT02300389: Comparing hypofractionated radiotherapy boost to conventionally fractionated (HYPOPROST)	Completed	2011–2019	Hypofractionated IMRT boost + ADT vs Conventional IMRT boost + ADT	-
NCT02472275: PLX3397, radiation therapy, and antihormone therapy for patients with intermediate- or high-risk prostate cancer	Completed	2015–2019	PLX3397 + RT + ADT (Leuprolide acetate, Goserelin acetate, or Degarelix)	-
NCT02229734: Fairly brief androgen suppression and stereotactic radiotherapy for high-risk prostate cancer – Protocol 2 (FASTR-2)	Completed	2014–2021	SBRT + LHRH (Leuprolide)	This innovative condensed treatment showed higher than expected late toxicities, and was terminated before phase 2 accrual [70]
NCT03311555: A salvage trial of AR inhibition with ADT and apalutamide with radiation therapy followed by docetaxel in men with PSA recurrent prostate cancer after radical prostatectomy (STARTAR)	Completed	2018–2022	Apalutamide + ADT + RT (salvage radiation therapy) + docetaxel	-
NCT03649841: Antiandrogen therapy, Abiraterone acetate, and Prednisone with or without neutron radiation therapy for patients with prostate cancer	Terminated	2020–2023	ADT + Abiraterone + Prednisone + RT vs ADT + Abiraterone + Prednisone	Terminated due to low accrual
NCT01439542: Fairly brief androgen suppression and stereotactic radiotherapy for high-risk prostate cancer (FASTR)	Terminated	2011–2017	Stereotactic radiotherapy + LHRH agonist	Higher than expected Grade 3 genitourinary/gastrointestinal toxicity
NCT02508636: Trial of radiotherapy with Leuprolide and Enzalutamide in high-risk prostate cancer	Terminated	2015–2020	Enzalutamide + Leuprolide + IMRT	Terminated due to low accrual
NCT01811810: Proton therapy for high-risk prostate cancer	Withdrawn	2013–2014	XRT + ADT vs XRT + chemotherapy + short-term ADT	Unable to recruit
NCT01517451: Radiation and androgen ablation for prostate cancer	Active	2013–2026	ADT + SBRT	-
NCT01952223: A phase III study of Cabazitaxel and pelvic radiotherapy in localized prostate cancer and high-risk features of relapse (PEACE2)	Active	2013–2041	ADT + pelvic RT ADT + Cabazitaxel + prostate RT ADT + Cabazitaxel + pelvic RT ADT + prostate RT	-
NCT01546987: Hormone therapy, radiation therapy, and steroid 17alpha-monooxygenase TAK-700 for patients with high-risk prostate cancer	Active	2012–2029	ADT + RT vs TAK-700 + ADT + RT	-
NCT04489745: Stereotactic body radiotherapy (SBRT) for localized prostate cancer	Active	2016–2025	ADT + SBRT	-
NCT03541850: Stereotactic body radiation therapy for patients with localized prostate cancer that have undergone surgery	Active	2019–2024	ADT + SBRT	-
NCT02346253: High-dose brachytherapy for patients with prostate cancer	Active	2015–2026	HDR brachytherapy + Bicalutamide + Leuprolide acetate + Goserelin acetate + Triptorelin pamoate + Degarelix	-
NCT00936390: Radiation therapy with or without androgen deprivation therapy for patients with prostate cancer	Active	2009–2025	EBRT vs. EBRT + ADT	-
NCT01436968: Phase 3 study of ProstAtak^®^ immunotherapy with standard radiation therapy for localized prostate cancer (PrTK03)	Active	2011–2024	ProstAtak^®^ + RT +/- ADT	-
NCT02594072: Androgen suppression with stereotactic body or external beam radiation therapy (ASSERT)	Active	2016–2024	SABR + Zoladex^®^ Vs. EBRT + Zoladex^®^	-
NCT02446444: Enzalutamide in androgen deprivation therapy with radiation therapy for high-risk, clinically localized prostate cancer (ENZARAD)	Active	2014–2025	Enzalutamide + LHRHA + EBRT	-
NCT01420250: Cabazitaxel with radiation and hormone therapy for prostate cancer	Active	2011–2023	Cabazitaxel + IMRT + Bicalutamide + LHRH agonist	-
NCT02531516: An efficacy and safety study of JNJ-56021927 (Apalutamide) in high-risk prostate cancer subjects receiving primary radiation therapy: ATLAS	Active	2015–2026	Apalutamide + Bicalutamide Placebo + GnRH (agonist) + RT	-
NCT03070886: Antiandrogen therapy and radiation therapy with or without docetaxel for patients with prostate cancer that has been removed via surgery	Active	2017–2031	ADT + EBRT Vs. ADT + EBRT + docetaxel	-
NCT05003752: Hypo-Combi trial: Hypofractionated EBRT plus HDR-BT boost for prostate cancer	Active	2021–2026	Hypofractionated EBRT + HDR-BT boost	-
NCT04947254: Androgen ablation therapy with or without niraparib after radiation therapy for the treatment of high-risk localized or locally advanced prostate cancer	Recruiting	2021–2026	Apalutamide + ADT, ADT + Abiraterone acetate and Prednisone, with or without Niraparib after RT	-
NCT04298983: Abemaciclib in combination with androgen deprivation therapy for locally advanced prostate cancer (RAD 1805)	Recruiting	2021–2026	Abemaciclib + ADT + RT	-
NCT05753566: Rezvilutamide in patients with biochemical recurrence after radical prostatectomy for prostate cancer	Recruiting	2023–2028	Rezvilutamide + ADT + SRT Rezvilutamide + ADT	-
NCT02303327: Comparative study of radiotherapy treatments for high-risk prostate cancer patients	Recruiting	2015–2029	ADT + EBRT + HDR brachytherapy boost ADT + hypofractionated dose-escalation RT	-
NCT05781217: Short- versus long-term androgen deprivation therapy with salvage radiotherapy in prostate cancer (URONCOR 06-24)	Recruiting	2023–2032	STADT + RT LTADT + RT	-
NCT05100472: A study of shorter-course hormone therapy and radiation for high-risk prostate cancer	Recruiting	2021–2024	ADT + brachytherapy + hypofractionated pelvic external beam radiation	-
NCT05361798: T-cell clonality after stereotactic body radiation therapy alone and in combination with the immunocytokine M9241 in localized high- and intermediate-risk prostate cancer treated with androgen deprivation therapy	Recruiting	2023–2024	De-escalating dose of M9241 + SBRT High tolerated dose of M9241 + SBRT SBRT	
NCT01985828: CyberKnife^®^ as monotherapy or boost SBRT for intermediate- or high-risk localized prostate cancer	Recruiting	2013–2026	ADT + CyberKnife + IMRT	-
NCT04943536: Bicalutamide implants (Biolen) with radiation therapy in patients with localized prostate cancer	Recruiting	2021–2024	Biolen + RT	-
NCT04894188: Neoadjuvant hormone and radiation therapy followed by radical prostatectomy in patients with high-risk prostate cancer	Recruiting	2022–2041	Neoadjuvant ADT and RT + radical prostatectomy Neoadjuvant ADT + radical prostatectomy	-
NCT05568550: Pembro with radiation with or without Olaparib	Recruiting	2023–2029	Pembrolizumab + Olaparib + ADT + RT Pembrolizumab + ADT + RT	-
NCT04349501: Biomarker monitoring of prostate cancer patients with RSI MRI (ProsRSI)	Recruiting	2020–2026	RSI-MRI + ADT + RT	-
NCT04136353: Darolutamide augments standard therapy for localized very-high-risk prostate cancer (DASL-HiCaP)	Recruiting	2020–2028	Darolutamide + LHRHA + EBRT	-
NCT02102477: Surgery versus radiotherapy for locally advanced prostate cancer (SPCG-15)	Recruiting	2014–2045	Radical prostatectomy + RT vs RT + adjuvant ADT	-
NCT04093375: Radical prostatectomy versus radical radiotherapy for locally advanced prostate cancer	Not yet recruiting	-	Radical prostatectomy +/- ADT vs RT + adjuvant ADT	-
NCT04176081: Study of radiation therapy in combination with Darolutamide + Degarelix in intermediate-risk prostate cancer (SChLAP/IDC)	Not yet recruiting	-	RT vs RT + Darolutamide + Degarelix	-

ADT = androgen deprivation therapy; RT= radiotherapy; LTADT= long-term ADT; STADT = short-term ADT; LHRHA = luteinizing hormone-releasing hormone analogue; SABR = stereotactic ablative radiotherapy; EBRT = external beam radiotherapy; SBRT = stereotactic body radiation therapy; IMRT = intensity-modulated radiation therapy; HDR-BT= high-dose-rate brachytherapy.

### 2.5. Use of a Radiosensitizer

RT is the most extensively used and effective antitumor therapy for cancer. While irradiation can effectively destroy cancer cells, it also damages normal tissues and cells near the treated area. Therefore, precision RT has emerged as a significant trend in the advancement of RT technology to reduce damage to normal tissues. The use of radiosensitizers (RSs) is becoming an important part in the development of precision RT.

RSs are drugs used to enhance the sensitivity of tumor cells to radiation, the increasing the efficacy of RT in killing tumor cells, leading to better control and cure rates and have been widely investigated in the past decade [71]. They are pharmaceutical agents that can expedite DNA damage and produce free radicals, intensifying the killing effect on tumor cells with a limited impact on normal tissues [72]. Several strategies have been adopted to develop RSs that are highly effective and maintain low toxicity levels [71,72]. RSs have been developed for decades from early strategies such as “free radical damage and fixation” strategies to gene regulation, from chemical agents to biological macromolecules and nanomaterials.

The main mechanisms involved were indirect and direct. The indirect mechanisms included (I) disruption of organelle function and cell cycle to increase cytotoxicity, (II) aggravation of DNA damage together with inhibition of radiation-induced DNA damage repair, and (III) promotion of the expression of radiation-sensitive genes or suppression of the expression of radiation-resistant genes [73,74]. RT may exert its effects on cancer cells by generating reactive oxygen species (ROS) through a physicochemical reaction between IR and RSs. RSs are currently being developed rapidly using nanotechnology, and cancerous tissues or cancer cells selective RSs play a crucial role in the future of precision RT. These RSs are studied to acquire sufficient understanding of their selectivity for cancer tissues.

The combination of gefitinib and three-dimensional conformal RT for treating nonmetastatic PCa was well tolerated by the patients and yielded promising results, with 97% of the patients showing no biochemical evidence of tumor recurrence after a median follow-up of 38 months [75]. We provide a summary of these clinical trials (Table 2). Other small phase II studies reported the combined use of maximum androgen blockade, bevacizumab, and concomitant RT for patients with high-risk PCa [76]. The treatment was well tolerated by the patients, with no increase in acute toxicities. However, a minor increase in late toxicities correlated with proctitis and cystitis was observed.

A small pilot study conducted by Ahmad et al. investigated the use of soy isoflavone or placebo in combination with EBRT in a patient with localized PCa. A total of 42 patients were randomly assigned to receive soy isoflavone or placebo with the use of total radiation (77.5 Gy) [77]. The results suggest that the therapy could be well tolerated and possibly beneficial in reducing radiation-related side effects, such as urinary, intestinal, and sexual adverse effects.

Curcumin (diferuloylmethane) is a polyphenolic active compound derived from turmeric. It exerts its anti-inflammatory effect by inhibiting the activity of transcription factor NF-κB, which is engaged in both radio resistance and tumorigenesis [78]. In a preclinical study, Chendil et al. reported that the PCa cells when treated with combination of curcumin with RT, there was a 3-fold fewer surviving cancer cells and inhibition of NF-κB activity was seen [79]. The potential of nanocurcumin to be used as an RS is being evaluated by a phase II clinical trial (NCT02724618). Another relevant study involving mutant p53 Ewing sarcoma cells found that the radiosensitivity of curcumin is associated with other p53 response genes, which may enhance the sensitivity of cancer cells [80].

The compound 5-aminolevulinic acid (5-ALA) leverages the unique metabolic processes of cancer cells to specifically accumulate protoporphyrin IX (PpIX). This compound has been used in clinical applications in photodynamic diagnosis and therapy, and selectively accumulated data conform to PpIX in various tumors. Furthermore, PpIX, as an RS, generates ROS upon exposure to radiation [81]. The efficacy of 5-ALA has been tested in different cancer types. Miyake et al. investigated the radioprotective ability of 5-ALA against normal tissues from radiation while enhancing the radiosensitivity of tumor cells [82].

Enzalutamide, classified as a second-generation antiandrogen known for its high affinity and activity, is used for the treatment of metastatic disease [83]. It has demonstrated radiosensitizing activity in both androgen-independent and androgen-dependent human PCa models across various experiments in cell culture studies, xenografts in mice, and treatment-resistant patient-derived xenografts. These findings support the use of Enzalutamide as an RS for the treatment of PCa [84]. Several ongoing clinical trials are investigating the combined use of Enzalutamide and RT in different groups of patients with intermediate-risk localized PCa (NCT02023463, NCT02028988, and ENZART NCT03196388) and high-risk localized PCa (ENZARAD NCT02446444, NCT02508636, and NCT02064582) and in patients with a history of prostatectomy and receiving salvage RT for persistent PCa (NCT02203695 and STREAM [NCT02057939]). These clinical trials provide key mechanistic data to support the enhanced tumor-killing effect of the combined use of Enzalutamide and RT, providing improved localized treatment options for patients with castration-resistant and hormone-sensitive PCa.

Several ongoing clinical trials are investigating the combined use of nanoparticle RSs and RT in patients with PCa such as a phase I/II clinical trial (NCT02805894) of hafnium oxide nanoparticles (NBTXR3) in PCa are under evaluation [74].

**Table 2:** A list of registered clinical trials of radiosensitizers for prostate cancer. ‘-’ denotes being not clear.

Trials	Radiosensitizer Used	Risk Group	Target	Trial Phase	Trial Period	Trial Status	Outcomes
NCT02724618	Curcumin+RT+Placebo	-	NF-κB	II	2016–2022	Completed	Hematologic toxicity as assessed by significant reduction in hematologic components. Biochemical progression-free survival (b-PFS).
NCT03066154	Docetaxel+ADT+RT	-	-	I	2016–2020	Completed	*C*_max_ was significantly lower for both docetaxel and ritonavir was significantly lower in prostate cancer patients as compared to patients with other types of solid tumours, treated on ModraDoc006/r 30-20/100-100 [85]
NCT02057939	Enzalutamide+ADT+ RT	High risk	Androgen receptor	II	2014–2019	Completed	Biochemical progression-free survival, PSA less than 0.1 ng/mL.
NCT00631527	Sunitinib + ADT	High risk	Multi-targeted RTK	I	2008–2015	Completed	-
NCT02023463	Enzalutamide+RT+ADT	High-Risk	androgen receptor	1	2014–2040	Recruiting	-
NCT02203695	Enzalutamide+SRT	High risk	androgen receptor	II	2015–2024	Recruiting	-
NCT02805894	NBTXR3 nanoparticles + brachytherapy + IMRT	Intermediate or high risk	prostate Adenocarcinoma	I/II	2017–2021	Recruiting	-
NCT00943956	Everolimus + ADT	High risk	mTOR	I	2009–2012	Unknown	-
NCT01826838	Dasatinib + ADT	Intermediate-to high-risk	SRC	I	2012–2022	Recruiting	-

## 3. Treatment Intensification vs. Active Surveillance

At present, many PCa patients in their 60s are diagnosed with localized, not metastatic, diseases. The realization that ADT, a common treatment approach, is not a curative treatment has led to the establishment of two alternative approaches to PCa treatment: (1) enhancing systematic therapies for advanced cases and (2) implementing rigorous screening methods for the early detection and localized treatment of PCa [9]. AS for PCa emerged as a response to the increased detection of indolent, low-risk PCa cases, mainly due to widespread PSA screening in the 1990s. It has been designed to combat overtreatment by delaying or circumventing unnecessary definitive treatments and its associated potential for treatment-related morbidity. AS involves regular monitoring of various factors, such as PSA levels, digital rectal exams, medical imaging, and prostate biopsies, to delay or avoid unnecessary definitive treatment. AS was initially performed in a study setting, but over the 25 years, numerous studies have since demonstrated its safety and efficacy, leading to its application as a standard care strategy in the management of low-risk PCa and patients with favorable intermediate-risk disease.

The convergence of improved patient selection and personalized, aggressive adjustable follow-up became feasible, and reduction in invasive monitoring holds great promise in decreasing the cases of overtreatment [86,87]. Two studies found that radical prostatectomy, EBRT + ADT, and AS resulted in very high survival rates, and no significant difference was observed in prostate testing for cancer or all-cause mortality between the treatment groups [88,89]. But the AS group was more likely to have bone metastasized PCa. 

AS and TI appear to be conflicting choices for PCa treatment. Because of the difficulty in predicting the progression status of PCa, an accurate choice will change the fate of the patients. Accordingly, criteria to stratify patients with more precision in the selection of treatment choices needs to be developed.

## 4. Complications

PCa therapy has been steadily moving toward TI over the past decade and is continuously evolving, but the road to it can encounter many common adverse events associated with the additional agents. A better understanding of the role of tumor markers of toxicity and response to RT in patients with PCa is definitely required for better outcomes. Side effects arise when the healthy tissues close to the prostate is damaged by RT [90]. The side effects of RT can differ depending on the category and dose of radiation as well as the specific area treated. Majority of the short-term side effects are temporary and tend to gradually improve weeks after the treatment completion. Of note, patients who undergo RT usually do not experience permanent effects on urinary or bowel function [91,92]. Patients who develop erectile dysfunction can often be successfully treated with sildenafil or tadalafil. POTEN-C trial (NCT03525262), a study randomizing standard vs. neurovascular-sparing CT guided EBRT, and ERECT trial (NCT04861194), which is a single-arm study delivering neurovascular-sparing radiotherapy through state-of-the-art adaptive MRI-guided EBRT are investigating preservation of erectile function with neurovascular sparing in patients with localized PCa [93]. 

Side effects that do not improve or occur months to years after pelvic RT are called long-term or late effects. As the prostate is located near numerous vital structures, RT can cause proctitis, narrowing of the rectum or urethra, urinary or rectal bleeding, cystitis, bowel inflammation, fatigue, sexual dysfunction, bone impairment, metabolic complications, cardiovascular events, and secondary cancer [16,91]. There is a reduced risk of side effects (such as bowel problems) thanks to a special hydrogel technology, which can shield the bowel during the treatment period. 

Observational studies demonstrated that brachytherapy and EBRT are associated with the risks of developing sexual, bowel, and urinary dysfunctions [94,95]. Patient-reported results indicated that men who received EBRT reported increased level of bowel disorder compared with the other groups, whereas erectile dysfunction occurred after around 6 months [96]. As regards urinary issues, EBRT causes urinary voiding and nocturia at the 6th month but showed significant recovery after 12 months to a level comparable to other treatment groups [96].

The recent result of PACE-B that high-dose RT improved cancer free rates within a fraction of the standard treatment time and less complications than traditional RT indicates a reduction in life-altering side effects of PCa from fewer visits to hospitals and less mental burden of treatment sessions [40].

PCa patients have an increased risk for cardiovascular complications. In a cohort of patients with intermediate- or high-risk localized PCa, high and intermediate calculated Framingham risk was were found in 65% and 33% of patients, respectively, putting focus on importance of cardiovascular concomitant disorder in this population group [97]. PCa therapies may accelerate or contribute to the development of potential cardiovascular complications such as cardiomyopathy, myocardial infarction, atherosclerosis, arrhythmia, stroke, and hypertension, along with other thromboembolic diseases [98]. While it is correlated with excellent PCa control results, hormonal changes from ADT causes cardiometabolic risk that can increase the rate of mortality from cardiovascular complications [99]. It is well established that ADT can result in detrimental metabolic transitions, such as insulin resistance, obesity and diabetes, metabolic syndrome, and cardiovascular disease. Clinicians should inform patients that PCa treatment poses risks in urinary, sexual, and bowel functions. In addition, the potential benefits of therapy against these risks should be carefully considered before initiating ADT. Further studies are warranted to determine the ideal strategy for selecting patients eligible for ADT and to identify the optimal strategy for the management of the side effects of ADT [100]. Concomitant ADT has been suggested to be possibly advantageous (at least in terms of metastasis-free survival) for PSA rates over 0.6 ng/mL, taking into account life expectancy and cardiovascular comorbidities [61].

The CHAARTED (NCT00309985) [101], STAMPEDE (NCT00268476) [102], and LATITUDE (NCT01715285) trials have demonstrated improved outcomes of the combination of standard ADT and different drugs or hormonal therapies [103]. However, some additional agents cause adverse effects, such as Abiraterone, which carries the risk of apparent mineralocorticoid excess that can lead to hypertension, low serum potassium level, swelling, and elevated liver functions; these effects can be avoided if patients are well monitored [103].

Do patients with PCa have a higher risk of developing a second primary cancer after RT compared with non-RT treatments? A recent cohort study of 143,886 patients with localized PCa showed that RT was associated with a small but statistically significant increase in the risk of a second primary cancer [104]. Another recent study found higher rates of bladder cancer after RT [105]. Shared decision making should include discussion of the risk of developing a second primary cancer and the possibility of a second primary cancer should be considered in the evaluation of PCa survivors after RT.

Immune monitoring of the 18 patients showed that RT can affect the balance of systemic immune cells, with the main differences observed between SBRT and conventionally fractionated RT [106]. While conventional RT had a long-term negative effect on the systemic immune profile, SBRT favorably impacts immune response in terms of increased B cells, central-memory and effector-memory CD8^+^ T cells, along with decreased Treg cells after treatment.

## 5. Role of Precision Medicine

Care for cancer patients has significantly improved, with the evidence-based medicine prototype guiding clinical decision making. However, the enormous heterogeneity of disease states, patients, and each patient’s environment is realized as it influences treatment implication and toxicities. Such heterogeneity compelled the reassessment of previously generalized randomized trials and increased interest in accounting for this heterogeneity within the criteria of precision medicine. Indeed, recent technological improvements and ongoing clinical research have provided radiation oncologists with personalized treatments for an accurate delivery of radiation dose based on both anatomical information and clinical parameters.

It has been suggested that two major strategies together will further increase the therapeutic potential of radiation oncology in the era of precision medicine: (1) new biological concepts for personalized treatment, including the use of biomarker-guided prescription, combination of different treatment modalities, and swift and correct adaptation of treatment during its course and (2) technology-driven enhancement of treatment precision, including advanced image guidance and use of particle therapy [8]. For this, it is important to develop critical informatics infrastructure on which precision RT can be established. The characterization and collation of all clinically relevant sources regarding heterogeneity that influences the long-term health outcomes of cancer patients undergoing RT provide a unique opportunity to establish a vital informatics infrastructure on which precision RT will be realized. It is expected that by leveraging locally developed yet coordinated informatics infrastructures, all of the discoveries of data science-driven insight, personalized clinical decisions, and the potential to accelerate translational efforts within the network of institutions will be realized [107]. The implementation of precision medicine will encourage radiation oncologists to establish personalized treatments based on clinical parameters and anatomical information.

Another important and necessary realization is genomics-based or genomics-guided RT. The mutational incidence of PCa is generally considered to be low compared with other epithelial tumors, but it seems to increase as the tumor becomes castration resistant and progresses to metastatic stages despite being initially treated with ADT and RT [108]. At the gene level, there are significant differences between localized and castration-resistant PCa. In localized PCa, molecular sequencing studies have often identified single-nucleotide variants with uncertain significance, and genetic mutations are detected in less than 10% of cases. In localized diseases, mutations in the AR are rarely observed, contrary to castration-resistant tumors that have progressed after ADT.

In a previous study of castration-resistant PCa, the tumors of 150 patients with metastatic castration-resistant PCa were sequenced [109]. The result indicated abnormalities in genes associated with the androgen signaling axis in 71.3% of cases, with the AR gene being the most commonly mutated gene. Additional mutations were also identified at various levels related to the androgen pathway. The findings allowed for the subclassification of castration-resistant PCa into different molecular subtypes, including AR, DNA repair, and PI3K–AKT pathways [108]. Gene mutations involved in homologous recombination repair induce different responses to various treatment schemes. For example, the presence of BRCA 1, 2 and ATM mutations does not reduce sensitivity to ADT. However, it decreases sensitivity to taxanes and increases sensitivity to platinum-based chemotherapy and to therapy using poly (ADP-ribose) polymerase inhibitors [108].

Prevalent genomic data that facilitate personalized RT, ongoing clinical trials, current challenges, and future directions were summarized [110]. The findings of NRG Oncology/RTOG 0126 validated the biopsy-based 22-gene Decipher genomic classifier in the risk stratum of patients with intermediate-risk PCa [111,112,113]. This was the first phase III trial authentication of any gene expression biomarker from pretreatment biopsy specimen in patients with intermediate-risk PCa treated with RT in the absence of ADT, shown to improvise risk stratification and can assist in treatment decision making in patients with intermediate-risk cancers [114]. These findings suggest that gene mutations determine therapeutic responses, including resistance, and that changes in gene mutation profiles acquired from treatment can be investigated to improve risk stratification and facilitate treatment decision making. 

In conclusion, the application of genomically guided RT is a critical step that must be embraced in the coming years. It seems apparent that there is substantial opportunity to fully apply genomically guided RT in the clinical setting.

## 6. Conclusions

PCa is a highly heterogeneous tumor with a wide range of clinical outcomes and morphological patterns, as well as some unusual metaplastic differentiations. The variability of PCa remains poorly understood. Nevertheless, spatial, genetic, and molecular heterogeneities, including interpatient, intertumoral, and intratumoral variabilities, are known to play pivotal roles in the disease pathogenesis. Also, it has been reported that, depending on the expertise of clinicians to whom PCa patients are referred, the selection of treatment schemes remains to be determined. In countries like South Korea, a higher number of PCa cases are diagnosed by urologists, which may lead to the preference of surgery as a treatment [115]. In Australia, it was found that few patients had consulted radiation oncologists before surgery and men are twice as likely to undergo radical prostatectomy as EBRT [116]. In both studies, differences also exist in the selection of primary treatment according to sociodemographic factors. Rapidly increasing cases of robotic surgery in many developed countries is indeed increasing total surgical volumes [117]. These indicate that the preferences for PCa treatment are not uniform across different countries.

Despite the improved OS, increased quality of life, and decreased disease progression, TI has not yet been applied in many patients. The National Data Surveys of US from 2015 to 2021 indicated that many urologists and oncologists continue to prescribe ADT alone. On average, 12% of men were prescribed chemotherapy and 25% with novel hormone therapy. Urologists prescribed novel hormone therapy as first-line treatment in only 12% of cases whereas oncologists did so in 32% of cases [118]. With regard to when to intervene with RT post-operatively, many in the radiation oncology community think treatment is more efficacious earlier in the disease process [119,120]. In contrast, many in the urology community defer referral of the patient to radiation oncology until PSA is continuously elevated [120,121,122,123]. 

The expertise and resources that accompany high-volume treatment facilities were shown to be associated with improved outcomes for men with very high-risk PCa [124]. In Korea, a nationwide pattern of care analysis showed the implementation of irregular radiation techniques and various dose fractionation schemes for PCa treatment [115]. The authors concluded that standard guidelines for RT in PCa cases need to be improved. It is unclear whether standard guidelines for RT in PCa are applied in many countries. Physicians in specific countries should reach a concurrence on the implementation of RT for PCa treatment worldwide.

Evidence suggests that intensification of treatment schemes seems promising. Despite strong data supporting the use of TI, it will still take time to put changes into practice due to concerns about side effects, data generalizability, tolerability, cost, and lack of awareness about the benefits associated with it. More research studies are needed to confirm its efficacy and direct applicability to patients from extensive yet well-coordinated informatics infrastructures that can realize genomics based risk stratifications in the long treatment courses of individual PCa patients as well as discoveries of data science-driven insight, personalized clinical decision making, and the potential to expedite translational efforts.
